# Evolutionary dependency of cancer mutations in gene pairs inferred by nonsynonymous-synonymous mutation ratios

**DOI:** 10.1186/s13073-024-01376-7

**Published:** 2024-08-19

**Authors:** Dong-Jin Han, Sunmin Kim, Seo-Young Lee, Youngbeen Moon, Su Jung Kang, Jinseon Yoo, Hye Young Jeong, Hae Jin Cho, Jeong Yang Jeon, Byeong Chang Sim, Jaehoon Kim, Seungho Lee, Ruibin Xi, Tae-Min Kim

**Affiliations:** 1 Department of Medical Informatics, College of Medicine, The Catholic University of Korea, Seoul, Korea; 2 Cancer Research Institute, College of Medicine, The Catholic University of Korea, 222 Bandodae-ro, Seocho-Gu, Seoul, Korea; 3 Department of Biomedicine & Health Sciences, Graduate School, The Catholic University of Korea, Seoul, Korea; 4 Department of Pharmacology, Yonsei University College of Medicine, Seoul, Republic of Korea; 5 School of Mathematical Sciences and Center for Statistical Science, Peking University, Beijing, China; 6 CMC Institute for Basic Medical Science, The Catholic Medical Center of The Catholic University of Korea, Seoul, Republic of Korea

**Keywords:** Gene pairs, Cancer mutations, dNdS ratios, Evolutionary dependency, Mutation contexts, Genetic dependency, Drug sensitivity

## Abstract

**Background:**

Determining the impact of somatic mutations requires understanding the functional relationship of genes acquiring mutations; however, it is largely unknown how mutations in functionally related genes influence each other.

**Methods:**

We employed non-synonymous-to-synonymous or dNdS ratios to evaluate the evolutionary dependency (ED) of gene pairs, assuming a mutation in one gene of a gene pair can affect the evolutionary fitness of mutations in its partner genes as mutation context. We employed PanCancer- and tumor type-specific mutational profiles to infer the ED of gene pairs and evaluated their biological relevance with respect to gene dependency and drug sensitivity.

**Results:**

We propose that dNdS ratios of gene pairs and their derived cdNS (context-dependent dNdS) scores as measure of ED distinguishing gene pairs either as synergistic (SYN) or antagonistic (ANT). Mutation contexts can induce substantial changes in the evolutionary fitness of mutations in the paired genes, e.g., *IDH1* and *IDH2* mutation contexts lead to substantial increase and decrease of dNdS ratios of *ATRX* indels and *IDH1* missense mutations corresponding to SYN and ANT relationship with positive and negative cdNS scores, respectively. The impact of gene silencing or knock-outs on cell viability (genetic dependencies) often depends on ED, suggesting that ED can guide the selection of candidates for synthetic lethality such as *TCF7L2*-*KRAS* mutations. Using cell line-based drug sensitivity data, the effects of targeted agents on cell lines are often associated with mutations of genes exhibiting ED with the target genes, informing drug sensitizing or resistant mutations for targeted inhibitors, e.g., *PRSS1* and *CTCF* mutations as resistant mutations to EGFR and BRAF inhibitors for lung adenocarcinomas and melanomas, respectively.

**Conclusions:**

We propose that the ED of gene pairs evaluated by dNdS ratios can advance our understanding of the functional relationship of genes with potential biological and clinical implications.

**Supplementary Information:**

The online version contains supplementary material available at 10.1186/s13073-024-01376-7.

## Background

While cancer genomes often harbor tens of thousands of somatic mutations, only a small fraction of these mutations, known as cancer driver mutations, are implicated in tumor initiation and progression [1, 2]. Selective advantages conferred by driver mutations leave genomic footprints as evolutionary consequences of positive selection and these footprints can help in identifying cancer driver mutations [1, 3]. Such genomic hallmarks of driver mutations include higher-than-expected mutation frequencies compared to neutral mutations that are relatively free of selection [4, 5]. In addition to mutation frequencies, assessing evolutionary selection in cancer genomes can be adopted for cancer driver discovery. One metric is the normalized nonsynonymous-to-synonymous (dN/dS or dNdS) ratio. The dNdS ratios exploit that the frequency of silent mutations can serve as proxy to model expected mutation frequencies. This ratio evaluates the type of selection (i.e., dNdS ratios > 1 and < 1 indicate positive and negative selection, respectively) and the magnitude of selective pressure on individual cancer genes or genomes that shape the mutational landscape of cancer genomes [6].

Multiple cancer driver mutations often co-exist in cancer genomes, and their coordinated actions may play important roles in cancer development and progression [7, 8]. Mutations in synergistic relationships will be co-selected, whereas those in antagonistic relationships or functional redundancies will be excluded, leading to co-occurrence (CO) and mutual exclusiveness (ME), respectively. Such gene pairs have been evaluated in terms of functionality, such as the synergistic relationship between *APC*-*KRAS* [9] and *SMAD4*-*KRAS* mutations [10]. ME gene pairs may explain the functional redundancies of cancer driver mutations with complementary functions [11, 12] but also indicate the potentially antagonistic relationships of mutations leading to synthetic lethality [13]. The functional relationship across multiple mutations adds additional complexity to the selection of biomarkers for targeted therapies and poses challenges in combined therapy, for example when combined mutations of functionally associated genes predict the resistance to targeted therapy [14]. Thus, the accurate identification of functional gene pairs and assessments of their relationship are important in precision oncology with potentially significant clinical relevance [6].

Previous methodologies to identify functional pairs of mutations have primarily relied on the genomic distribution of gene pairs such as CO and ME gene pairs as reviewed elsewhere [15]. Alternative approaches such as network-based identification of recurrently mutated subnetworks and gene pairs are also available [16–18]. Given the utility of evolutionary measures in identifying singleton cancer drivers [3], evolutionary relationships might offer quantitative means to assess the presence and potential functionality of gene pairs in cancer genomes. However, it is still challenging to determine which evolutionary metrics can be used to infer the functional relationship of mutations in gene pairs.

In this study, we postulated that the relationships of mutations in gene pairs might represent evolutionary dependency (ED). This implies that a mutation occurring in a gene (as a mutation context) confers positive or negative selective pressure on the other mutations in the genes of the functional relationship. To quantify the level of selective pressure, we employed dNdS ratios assuming that dNdS ratios of a gene measured in the presence or absence of the mutations of other genes in the paired relationship (as mutation contexts), reflect the ED of the corresponding gene pairs. Using PanCancer and tumor type-specific mutational profiles of the Cancer Genome Atlas (TCGA) consortium [19], we evaluated the pairwise relationships of gene pairs in terms of ED. We first tested whether dNdS ratios and their derived cdNS (context-dependent dNdS ratios) scores can distinguish types of functional ED of known functional gene pairs. Gene pairs exhibiting synergistic (SYN) and antagonistic (ANT) relationships (with cdNS scores > 0 and < 0, respectively) demonstrated a good agreement with CO-ME gene pairs identified based on genomic distribution. Furthermore, we integrated data of cell essentiality and pharmacological perturbations in cancer cell lines to assess the impact of ED on genetic dependency and sensitivity to targeted agents.

## Methods

### Mutations

We obtained somatic mutation calls for over 10,000 tumor specimens from the TCGA consortium (“mc3.v0.2.8.PUBLIC.maf.gz”; https://gdc.cancer.gov/about-data/publications/pancanatlas) available at the GDC (Genomic Data Commons portal) website [19, 20]. The coordinates of mutations are based on human reference genome GRCh37/hg19. The consequences of mutations on amino acid residues encoding and tumor-normal (alt. and ref. counts) allele frequencies of individual mutations were also obtained from the same resources. As an independent resource, mutational profiles of 10,000 patients identified by panel-sequencing (MSKCC-IMPACT data) were obtained along with patient outcome data (overall survival) via cBioPortal (https://www.cbioportal.org/study/summary?id=msk_impact_2017) [21]. We used a reference set of gene pairs with functional relationship, obtaining 517 gene pairs (referred to as “Mut-Mut” interactions in the literature) via the SELECT algorithm [22]. We also gathered information on two specific categories of gene pairs, CO and ME (co-occurring and mutually exclusive, respectively), from the same datasets. For tumor type-specific ED analysis, we restricted the mutational profiles to those occurring in 20 tumor types with over 500 cases each. The abbreviated tumor types include bladder urothelial carcinoma (BLCA, *n* = 412), breast invasive carcinoma (BCRA, *n* = 1097), cervical squamous cell carcinoma (CESC, *n* = 307), colorectal adenocarcinoma (COAD/READ, *n* = 629), esophageal carcinoma (ESCA, *n* = 185), head and neck squamous carcinoma (HNSC, *n* = 528), kidney renal clear cell carcinoma (KIRC, *n* = 537), kidney renal papillary cell carcinoma (KIRP, *n* = 291), brain lower-grade glioma (LGG, *n* = 515), glioblastoma multiforme (GBM, *n* = 596), liver hepatocellular carcinoma (LIHC, *n* = 377), lung adenocarcinoma (LUAD, *n* = 522), lung squamous cell carcinoma (LUSC, *n* = 504), ovarian serous cystadenocarcinoma (OV, *n* = 587), pancreatic adenocarcinoma (PAAD, *n* = 185), prostate adenocarcinoma (PRAD, *n* = 500), sarcoma (SARC, *n* = 261), skin cutaneous melanoma (SKCM, *n* = 470), stomach adenocarcinoma (STAD, *n* = 443), and uterine corpus endometrial carcinoma (UCEC, *n* = 548). COAD-READ and LGG-GBM were merged for the analysis unless indicated. Microsatellite instability (MSI) status of MSI-H (high), MSI-L (low), and MSS (microsatellite stable) as well as the LGG subtypes of astrocytoma, oligoastrocytoma, and oligodendroglioma were obtained from the publications [23, 24].

### ED assessment with dNdS ratios and cdNS scores

**Fig. 1:**
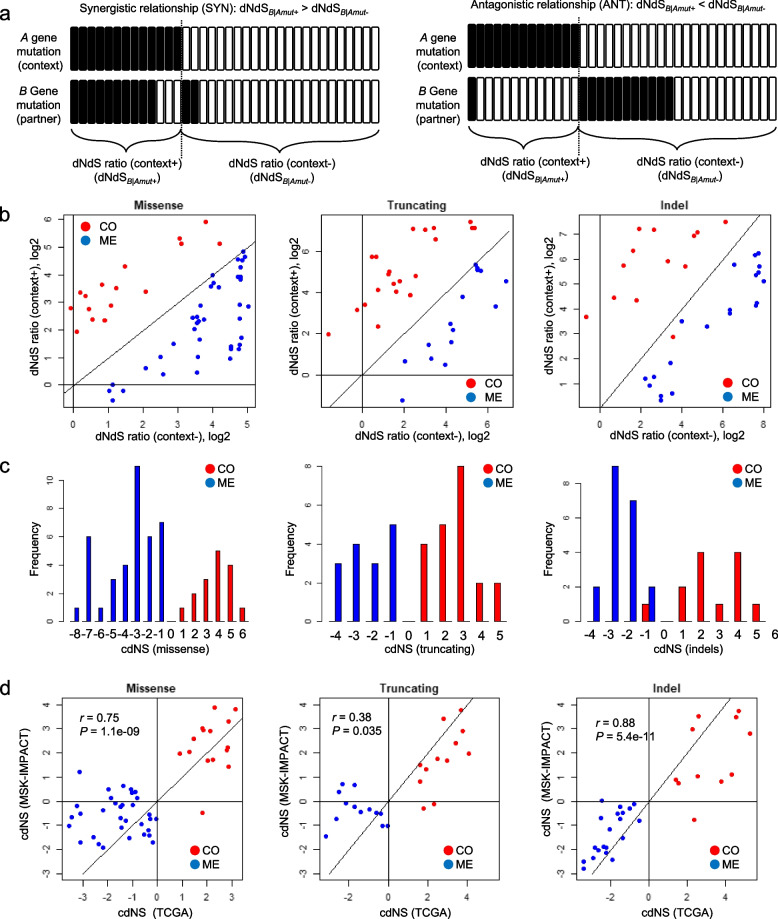
Schematics of dNdS ratio-based evolutionary dependency (ED) for mutation pairs. **a** Examples of synergistic (SYN) and antagonistic (ANT) gene pairs are shown for their presentations of mutations (depicted as the relationship between gene A and B). Two dNdS ratios (dNdS, context + and dNdS, context −) are calculated for gene B mutations in the presence and absence of mutation contexts (gene A mutations considered as mutation contexts). **b** Two dN/dS ratios are shown for known gene pairs with segregating gene pairs reflecting the types of functional relationship (red and blue for gene pairs representing co-occurrences (CO) and mutual exclusivity (ME), respectively). Three types of dN/dS ratios from missense, truncating, and indel mutations are shown separately, for those with significant ED (61 missense, 47 truncating mutations, and 41 indels, *P* < 0.01, permutation tests). **c** Summarized log2 odds of dN/dS ratios are calculated as cdNS (context-dependent dNdS ratios) scores and shown for missense, truncating mutations and indels. **d** cdNS scores of known gene pairs are compared with those obtained from independent MSK-IMPACT mutational profiles (TCGA and MSK-IMPACT, *x*- and *y*-axis, respectively)

The dNdS ratios were calculated using dNdScv R packages (https://github.com/im3sanger/dNdScv) using the genome version of GRCh37/hg19, which is compatible with those of mutational profiles [6]. To assess the level of ED for gene pairs (e.g., gene A and gene B), we determined the dNdS ratios of gene B in two different mutation contexts: with and without mutations of gene A. This led to two distinct dNdS ratios: dNdS_B|Amut+_ (with gene A mutations) and dNdS_B|Amut-_ (without gene A mutations), or simply, dNdS,context + and dNdS,context − , respectively. As mutation contexts (gene A mutations), we considered non-silent mutations occurring in the gene A, dividing the genomes into those with (mutation contexts) and without these mutations. We either directly compared these two dNdS ratios through scatter plots or combined them as log-odds to form a cdNS (context-dependent dNdS ratios) score (as shown in main Fig. 1b, c, respectively). The cdNS score, calculated as the log2 ratio of the dNdS ratio, context + to dNdS ratios, context − , serves to depict both the direction (e.g., mode the selection, SYN or ANT) and the magnitude of ED. We estimated the dNdS ratios and cdNS scores across three types of mutations: missense, truncating (nonsense and splice-site mutations), and indels, based on the dNdScv output.

To discover novel gene pairs exhibiting mutation-based ED, we selected a set of 312 genes. This comprised 220 genes identified in PanCancer mutational profiles (as determined by dNdScv with *q_global* < 0.1) and additional 92 genes that showed significant ED across tumor type-specific mutational profiles (significant in at least one tumor type). Details on these 312 genes and the tumor types analyzed are provided in Additional file 1:Table S1. The 312 genes served as mutation contexts, wherein dNdScv was employed to analyze genomes with and without their individual mutational contexts, utilizing the default set of 20,092 genes provided by the packages. The cdNS scores of individual gene pairs were calculated as the log2 ratio of two dNdS ratios (context + /context −), and their statistical significance was assessed via permutation tests unless otherwise specified.

### Statistical evaluation of gene pairs

The significance of cdNS scores was determined through permutation tests, where we shuffled the mutation context labels of genomes to generate a set of permuted dNdS ratios, context + and dNdS ratio, context − along with their cdNS scores for each gene pair. The significance was then assessed based on the frequency with which the permuted cdNS scores (1000 permutation tests) exceeded the actual score, providing nominal *P* values. In this exploratory analysis, we set the significance cutoff of gene pairs to be *P* < 0.01. To alleviate the tumor type-specific influence on the identification of gene pairs exhibiting ANT relationship in PanCancer analyses, we employed previously proposed sample size correction methods [25]. As previously described, a correction factor *w* was employed to reduce the number of mutation-negative genomes by rescaling the original denominator *N* (the total number of genomes). The correction factor *w* was calculated using sigmoid functions with recommended parameters [25]. We also used Fisher’s exact test to calculate the significance level for gene pairs in ANT relationship in PanCancer analysis by using the corrected sample sizes instead of permutation tests.

For power analysis, we selected two known gene pairs of *KRAS-STK11* synergistic (SYN) pair and *KRAS-NRAS* antagonistic (ANT) pair. To test the subsampling effect, we first selected 138 *STK11*-mutant and 272 *NRAS*-mutant genomes in PanCancer dataset as genomes with mutation contexts. Then, we subsampled varying number of these mutant genomes (ranging from 1 to 100 genomes), performing 100 subsampling for each group. We then calculated dNdS ratios (mutation context +) of *KRAS* missense mutations in subsample dataset to compare with dNdS ratios (mutation context −) to derive cdNS scores of *KRAS-STK11* and *KRAS-NRAS* pairs (Additional file 2: Figure S1a and S1b for details). The number of *KRAS* missense mutations in each permutation is also counted in subsampled datasets to use as proxy to determine the minimum number of mutations. Consistent cdNS scores were observed in at least 10 *STK11* and 50 *NRAS* mutant genomes where 3 and 1 *KRAS* missense mutations were observed. Based on this, we set a global, minimum threshold of mutations for SYN pairs (≥ 3 mutations) and ANT pairs (≥ 1 mutation). We also confirmed the cdNS values were relatively stable for these two gene pairs, even when the number of context − genomes was reduced to match that of the context + genomes (Additional file 2: Figure S1c). Additionally, we investigated the robustness of cdNS values with respect to the expression levels of mutation contexts. For this, the genomes with mutation contexts were divided into two categories: those whose genes of mutation contexts were expressed above the median level and those not (context-expressed and context-nonexpressed, respectively). For 85 gene pairs identified in PanCancer analyses (main Fig. 1), cdNS scores were calculated for genomes with context-expressed and context-nonexpressed. A significant correlation was observed between these two types of cdNS scores, indicating that the cdNS evaluation is relatively robust to the expression levels of mutation contexts (Additional file 2: Figure S1d).

Finally, SYN pairs in PanCancer data as well as SYN/ANT tumor type-specific pairs were evaluated for statistical significance by permutation tests. In PanCancer ED analyses, we selected gene pairs that showed significant ED (*P* < 0.01, permutation tests for PanCancer SYN pairs and *P* < 0.01, Fisher’s exact test for PanCancer ANT pairs), with a minimum number of mutations (SYN pairs ≥ 3 and ANT pairs ≥ 1), and ED ratios (dNdS context + /dNdS context −  > 3 or < 0.3) resulting in 85 gene pairs in PanCancer dataset. For tumor type-specific gene pairs, we also used the same criteria of PanCancer gene pairs (*P* < 0.01 in permutation tests both for SYN and ANT pairs, minimum number of mutations and ED ratios) identifying 3870 gene pairs across 20 tumor types.

### Mutation features

Variant allele frequencies (VAF) were determined by dividing the count of altered (alt. counts) alleles by the total number of reads (alt. counts + ref. counts) at the corresponding genetic loci. VAFs were computed separately for each gene of tumor type-specific gene pairs and gene-wise summarized as median values in corresponding tumor types. We then calculated the difference in VAF between two genes in each pair, by subtracting the median VAF of the context genes (gene A) from the median VAF of genes associated with the contexts (gene B). Thus, we expect the negative difference in VAF values (ΔVAF) when the mutations in context genes and their partner genes are clonal and subclonal, respectively, and positive values for vice versa. The ΔVAF were compared between SYN and ANT gene pairs across tumor types examined. For association with tumor mutation burdens (TMB), we considered the number of non-silent mutations in each genome. For each gene pair, we calculate the TMB of genomes harboring both mutations in the gene pairs and those only harboring mutations on one gene of the pairs. The differences in TMB (ΔTMB) were also compared between SYN and ANT gene pairs in a tumor type-specific manner. For the baseline distribution of ΔVAF and ΔTMB, we permuted the genes across the 85 PanCancer gene pairs. We conducted a hundred permutation tests to obtain the baseline distributions of ΔVAF and ΔTMB, then compared them with those of the SYN and ANT gene pairs.

### Relationship of ED and gene dependency

Gene dependencies of cancer cell lines were obtained from three databases: ANANA [26], DEMETER2 [27] and DRIVE [28] as indicated previously [22]. For each database, we estimated the genetic dependencies of gene pairs by calculating effect size with respect to mutation contexts. For instance, in the case of *KRAS-NRAS* gene pairs, where *NRAS* mutations represent mutation contexts, we collected cell lines containing *KRAS* missense mutations along with *KRAS* gene knockouts. These cell lines were then categorized into two groups: those with *NRAS* context mutations and those without. The effect size was determined as the difference in the median of cell viability between these two groups, aiming to evaluate whether *NRAS* context mutations impact KRAS knockouts in *KRAS*-mutated cell lines, either enhancing or compromising the cell viability. We then compared the effect size with their cdNS values as calculated in PanCancer or tumor type-specific mutational profiles. We also obtained gene pairs that appeared more than once across three databases of genetic dependency and calculated their average of effect size. Then, we classified gene pairs as either “compromising” or “rescuing,” determined by negative and positive effect sizes, respectively. A negative effect size (compromising) suggests that the presence of mutation contexts diminishes cell viability, while a positive effect size (rescuing) indicates an increase in cell viability. When combined with SYN-ANT ED types of gene pairs, this results in four categories: compromising-SYN/-ANT and rescuing-SYN/-ANT gene pairs. We also assessed pathway-level concordance of gene pairs by examining the frequency of occurrence of both genes across functional gene sets, utilizing Gene Ontology terms or gene sets from the MSigDB database (c5 gene sets as available in https://www.gsea-msigdb.org/gsea/msigdb) [29]. The occurrences of gene pairs in GO terms were *z*-normalized using the mean and standard deviation derived from permuted GO terms, where genes were randomly assigned across GO terms. Furthermore, we incorporated classifications of oncogenes and tumor suppressor genes as classified in the database of Cancer Census Genes for available genes [30].

### Pharmacology data

Drug response data for human cancer cell lines were obtained from GDSC (Genomics of Drug Sensitivity in Cancer) database [31]. From a combined set of two dataset versions (GDSC1 and GDSC2), the IC50 (half maximal inhibitory concentration) values were obtained across cell lines with their mutations and other clinical features such as tumor types (https://www.cancerrxgene.org/). We focused on well-established target agents (EGFR and BRAF inhibitors) in two tumor types of lung adenocarcinomas (LUAD) and skin melanomas (SKCM). The cdNS scores of gene pairs involving *EGFR* and *BRAF* mutations were collected from LUAD and SKCM tumor type-specific data. For drug sensitivity data, we obtained IC50 values of cell lines with respect to the mutations of target genes (e.g., *EGFR* and *BRAF* mutations) and their ED-associated genes (e.g., *KRAS* and *NRAS* mutations, respectively). To extend analyses of the relationship between ED and drug response, we collected IC50 values of drugs whose targets correspond to target mutations (e.g., EGFR and BRAF inhibitors) obtaining combinations of tumor type-drug-gene pairs (e.g., lung adenocarcinomas/LUAD – gefitinib – *EGFR/KRAS* pairs). This facilitated the comparison of drug sensitivity to target gene mutations (e.g., *EGFR* mutations) relative to mutation contexts (e.g., *KRAS* mutations) using effect size measurements. Thus, effect size was computed as the difference between IC50 values of cell lines with and without mutation contexts, both harboring the mutations corresponding to targeting agents and tumor types [31], e.g., IC50 of *EGFRmt/KRASmt* minus IC50 of *EGFRmt/KRASwt* LUAD cell lines. The effect sizes were also obtained from CCLE (Cancer Cell Line Encyclopedia) databases (https://sites.broadinstitute.org/ccle/) [32] for gene pairs supported by both datasets.

## Results

### dNdS ratios and cdNS scores as measures of ED of gene pairs

Given that multiple gene mutations are unlikely to occur simultaneously in a single cell, the selective pressure of paired gene mutations can be dissected into those imposed on the first and second mutation hits of the gene pair. The evolutionary pressure imposed on the second gene mutations in the presence of the first gene mutations is the major determinant of selective fitness of paired gene mutations leading to genomic footprints such as CO and ME gene pairs in cancer genomes. The first mutation establishes a mutation context that either supports or opposes the second mutation, based on their functional relationship. In this study, we adopted dNdS ratios to evaluate and quantify the levels of selective pressures on mutations in gene pairs. Specifically, we compare dNdS ratios for a second gene mutation in the context of an existing or absent first mutation, with schematics illustrated in Fig. 1a. Originally, dNdS ratios identified positive and negative evolutionary pressures (dNdS ratios > 1 or < 1, respectively) indicative of oncogenes and tumor suppressor genes (6), but can also distinguish between synergistic and antagonistic gene mutations pairs by considering mutation contexts. For instance, the dNdS ratios for gene B are evaluated in the presence of (dNdS_B|Amut+_) and absence (dNdS_B|Amut−_) of mutations in its partner gene A. A higher ratio (dNdS_B|Amut+_  > dNdS_B|Amut−_) suggests a synergistic relationship, favoring gene B mutations when gene A is mutated. Conversely, a lower ratio (dNdS_B|Amut+_  < dNdS_B|Amut−_) indicates either antagonism or functional redundancy. The pairs are termed “synergistic” (SYN, dNdS_B|Amut+_  > dNdS_B|Amut−_) and “antagonistic” (ANT, dNdS_B|Amut+_  < dNdS_B|Amut−_), respectively. These ED-based annotations of SYN/ANT are distinguished from CO and ME gene pairs inferred from genomic distribution. We define mutation contexts (mutations of gene A) as those that either advantageous or disadvantageous in the acquisition of functionally related gene mutations (gene B), thereby determining the selective fitness of gene A-B pairs. Also, we use dNdS ratios (context +) and dNdS ratios (context −) as a concise representation of dNdS_B|Amut+_ and dNdS_B|Amut−_, respectively.

As a proof-of-concept, we first examined known gene mutation pairs previously identified as CO or ME pairs (333 CO and 184 ME pairs) as available in a previous report (22). We calculated dNdS ratios for each gene in a pair considering the mutations in its partner gene’s mutations as mutation context. The analysis was conducted for three types of mutation categories: missense, truncating (nonsense and splice site), and indels, using PanCancer mutational profiles of TCGA consortium. Scatter plots demonstrate dNdS ratios for three types of mutation categories, with the *x*- and *y*-axis displaying dNdS ratio (context −) and dNdS ratio (context +) for 61 missense, 47 truncating, and 41 indel mutation gene pairs with significant ED (*P* < 0.01, permutation tests, Fig. 1b). The majority of genes showed dNdS ratios greater than 1 (log2 of dNdS ratio > 0), either with or without mutation context, suggesting that most genes in these pairs are likely cancer drivers under positive selection. Notably, CO and ME pairs (red and blues dots, respectively) tend to segregate along the diagonal line implying the types of selective pressure conferred on gene pairs (i.e., SYN and ANT pairs, respectively) are concordant with CO and ME genomic presentation of gene pairs. Compared with dNdS ratios of unfiltered 517 gene pairs (Additional file 2: Fig. S2a), gene pairs that located near the diagonal lines were mostly filtered based on their significance suggesting a substantial variability of ED for gene pairs previously categorized based on genomic distribution. Discordant gene pairs between two types of annotations (e.g., SYN-ME and ANT-CO pairs) were further examined regarding their level of enrichment for cancer-related genes (i.e., Cancer Census Genes) and compared with those of concordant gene pairs (e.g., SYN-CO and ANT-ME) (Additional file 2: Fig. S2b). The enrichment analysis suggests that concordant pairs are more enriched to cancer-related genes than discordant pairs. This indicates that the selective pressure conferred on cancer-related genes are likely to result in their anticipated genomic consequences.

We then introduced the “context-dependent dNdS ratios (cdNS),” determined by the log2 transformed ratio between the dNdS ratio (context +) and the dNdS (context −). This approach consolidates the two dN/dS ratios into a single value, effectively capturing the ED of gene mutation pairs. The cdNS values for missense, truncating, and indel mutation pairs (149 significant pairs in Fig. 1b) are depicted in Fig. 1c. The cdNS values were also able to distinguish between the CO and ME gene pairs across the three mutation types again highlighting the overall concordance of the SYN-ANT relationship with CO-ME genomic representation of gene pairs.

To further validate our findings, we compared cdNS values of known gene pairs estimated using PanCancer mutational profiles with those from independent cohorts of MSK-IMPACT cohorts [21]. Among 517 known gene pairs, 133 pairs were available for mutation counts in the MSK-IMPACT dataset and we observed similar trends in segregating CO and ME gene pairs based on cdNS score, as shown in Additional file 2: Fig. S3. Furthermore, the cdNS values for 48 missense, 31 truncating, and 32 indel pairs whose ED are both significant in two datasets (TCGA and MSK-IMPACT) were significantly correlated suggesting that the functional relationships of gene pairs are consistent across databases (Fig. 1d). For example, cdNS scores of missense mutations of TCGA and MSK-IMPACT were significantly correlated (*r* = 0.75, *P* = 1.1e − 09) along with truncating mutations (*r* = 0.38, *P* = 0.035) and indels (*r* = 0.88, *P* = 5.4e − 11). These findings indicate that the ED-based SYN-ANT relationship of gene pairs remains consistent across various mutation databases, thus serving as a reliable indicator of their functionality and evolutionary relationship.

### Identification of gene mutation pairs with ED

**Fig. 2:**
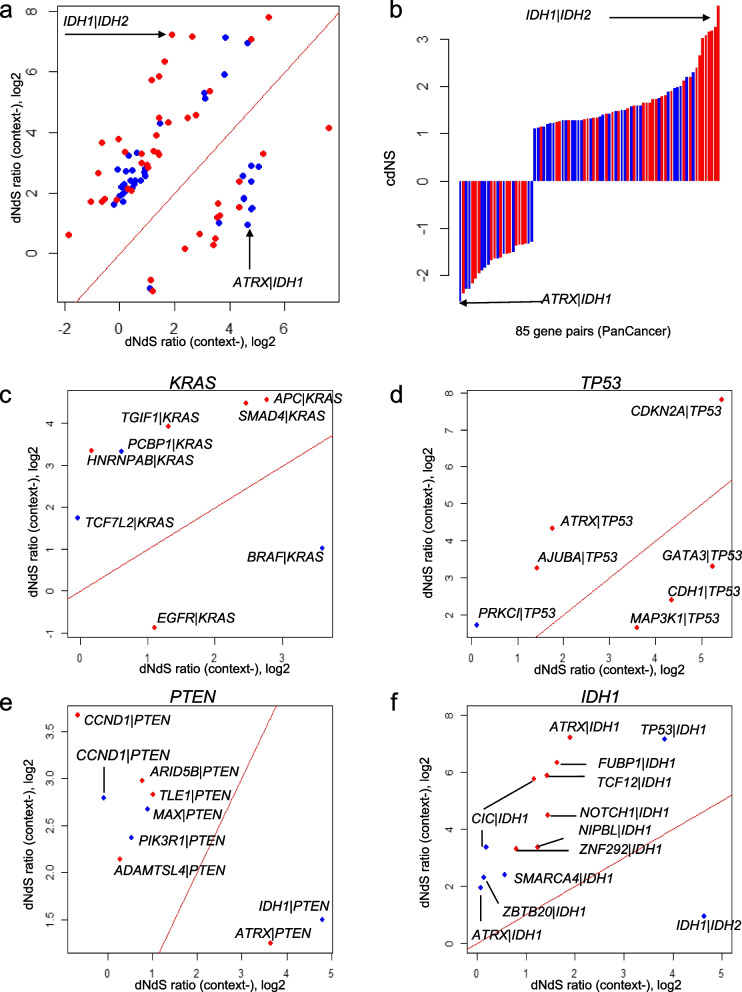
PanCancer gene pairs under ED. **a** A total of 85 gene pairs identified in PanCancer mutational profiles are displayed for their dNdS ratios. The *x*-axis represents the dNdS ratios with mutation contexts (context +), while the *y*-axis represents the dNdS ratios without mutation contexts (context −). Blue and red dots denote missense and indel gene pairs, respectively. **b** cdNS scores of 85 gene pairs are shown in order of the cdNS scores. Blue and red lines represent the missense and indel gene pairs, respectively. **c–f** The dNdS ratios in the presence and absence of mutation contexts are separately illustrated in scatter plots for *KRAS*, *TP53*, *PTEN*, and *IDH1* mutations. The *y*-axis represents the dNdS ratios with mutation contexts, while the *x*-axis represents the dNdS ratios without mutation contexts. Blue and red dots indicate missense and indel pairs, respectively

To identify gene pairs exhibiting ED, we selected a set of 312 genes composed of 220 genes and 92 genes identified from PanCancer and tumor type-specific mutational profiles, respectively (Additional File 1: Table S1). These genes exhibited significant overlap with known cancer-related genes [30], with 148 of them overlapping with a set of 719 known cancer-census genes. Additionally, 155 of these genes overlapped with 248 genes previously identified in known 517 gene pairs (where 248 gene members formed 517 gene pairs). We performed cdNS analyses for possible gene pairs of 312 genes using PanCancer mutational profiles. To assess the significance of cdNS scores, we employed permutation tests and implemented supplementary filters to account for tumor types and sample sizes (for detailed methods, refer to “Methods”). This analysis revealed 85 gene pairs with statistically significant ED, consisting of 41 missense gene pairs and 44 indel gene pairs. Among the pairs, 24 out of the 85 identified pairs (28.2%) overlapped with the 517 known gene pairs. For identified pairs, the dNdS ratios with or without mutation contexts are shown in a scatter plot (Fig. 2a) along with a barplot representing cdNS scores of the pairs (Fig. 2b). A comprehensive list of 85 gene pairs, along with related details can be found in Additional File 1:Supplementary Table S2. We observed that the smallest cdNS value was observed with *IDH1* missense mutations in the presence of *IDH2* mutation context (cdNS =  − 2.56) and the highest cdNS value was noted for *ATRX* indels with *IDH1* mutation contexts (cdNS = 3.69), indicative of ANT and SYN relationship, respectively (arrows indicated, Fig. 2a, b). This finding suggests that *IDH1* missense mutations occur approximately four times less frequently in genomes with *IDH2* mutations compared to genomes without *IDH2* mutations, indicating a potential antagonistic relationship or functional redundancy observed in brain tumors [33]. On the other hand, *ATRX* indels are found approximately ten times more frequently in genomes with *IDH1* mutations compared to those without *IDH1* mutations. This implies that *ATRX* losses, which lead to an alternative lengthening of telomeres, may not be sufficient on their own to drive tumor formation but may complement mutant *IDH1* expression, suggesting their potential functional synergism [34].

To further evaluate the impact of mutation contexts, we investigated four specific mutation contexts—*KRAS*, *TP53*, *PTEN*, and *IDH1*—that exhibited significant effects on gene pairs, with each occurring in 8, 7, 9, and 12 gene pairs, respectively. In the case of *KRAS* mutation contexts, we found that the dNdS ratios of eight gene pairs were substantially altered with *KRAS* mutations, indicating a functional association with *KRAS* mutations (Fig. 2c). For instance, the dNdS ratios of *BRAF* and *EGFR* mutations decreased (from 3.6 and 1.1 to 1.0 and − 0.9, respectively, on a log2 scale) in the presence of *KRAS* mutation contexts, suggesting an ANT relationship with *KRAS* mutations. Furthermore, *SMAD4* and *APC* indels exhibited a SYN relationship with *KRAS* mutations, consistent with previous reports [35, 36]. Other mutations, such as *TGIF1* loss, exhibited a SYN relationship with *KRAS* mutation contexts, consistent with their role in accelerating Kras-driven malignant transformation in the pancreas [37]. We also examined the impact of *NRAS* and *HRAS* mutation contexts on genes affected by *KRAS* mutation contexts using available cdNS scores for *NRAS* and *HRAS* mutation contexts (Additional File 2: Supplementary Fig. S4). Consistent observations of an ANT relationship with *BRAF* mutations (observed for both *NRAS* and *HRAS* mutation contexts) and a SYN relationship with *APC* mutations (for the *NRAS* mutation context) suggested that the ED relationship is largely preserved across members of the Ras gene family.

For *TP53* mutation contexts (Fig. 2d), genes like *ATRX* encoding SWI/SNF family chromatin remodeling proteins known to cooperate with p53 deficiency, showed elevated dNdS ratios, indicating synergistic roles [38]. Additionally, previously reported synergistic gene pairs, such as *CDKN2A-TP53*, were observed as SYN pairs [25]. Among genes exhibiting SYN relationship with *TP53* mutations, *AJUBA* and *PRKCI* have been reported to interact directly with p53 [39] and cooperate with *TP53* losses across various tumor types [40], respectively. Conversely, as genes showing an ANT relationship with *TP53* mutation contexts, *GATA3* mutations have been previously reported for the synthetic lethality of *GATA3* and *MDM2* in breast cancers [41]. In addition, the *CDH1* losses are also known to promote tumorigenesis with *TP53* losses in endometrial cancers [42]. Our analyses also revealed a number of *PTEN*-synergistic mutations such as *PIK3R1* and *CCND1*, e.g., *PIK3R1* missense mutations dNdS ratios from 0.53 to 2.39 and *CCND1* indels dNdS ratios from − 0.66 to 3.67, respectively with *PTEN* mutation contexts (Fig. 2e). Among the members in a phosphatidylinositol 3-kinase (PI3K) pathway, mutations of *PIK3R1* disrupt the genome stabilizing roles of *PTEN*, potentially synergizing *PTEN* losses in endometrial cancers [43]. *CCND1* overexpression has been often observed with *PTEN* alterations in lung cancers suggesting SYN relationship [44]. Although *CCND1* amplification has been proposed as major alterations in cancer genomes, we observed both missense mutations and indels of *CCND1* were associated with *PTEN* mutation contexts. In addition, the ANT presentation of *PTEN* and *IDH1* mutations have been previously recognized for some tumor types such as gliomas [45]. Genes with synergistic relationship with *IDH1* mutations in glioma such as *ATRX* have been previously reported [34], representing a glioma subgroup distinct from those with co-mutations of *IDH1* with *CIC* and *FUBP1* mutations [46] (Fig. 2f). Synergistic relationship of missense and indel *TP53* mutations were observed within *IDH1* mutation contexts, consistent with the known relationship in brain tumors where up to 70% of *IDH1*-mutant astrocytomas harbor *TP53* mutations [47]. *IDH2* ANT relationship suggests functional redundancy with *IDH1* mutations in glioma pathogenesis [34].

### Identification of genes pairs with tumor type-specific ED

**Fig. 3:**
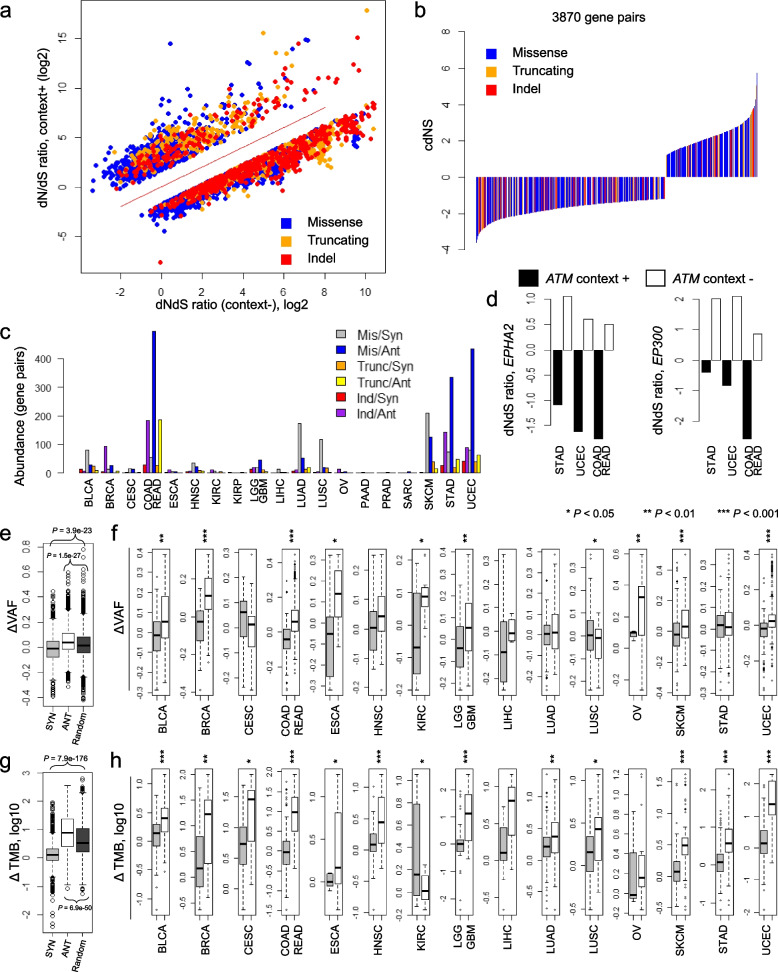
Tumor type-specific gene pairs under ED. **a,b** A total of 3870 mutation pairs gene pairs exhibiting tumor type-specific ED are depicted, showcasing their dNdS ratios and cdNS scores. **c** The prevalence of tumor type-specific gene pairs are shown across the examined tumor types. Colors represent the ED types (SYN and ANT) and mutation types (missense, truncating mutations, and indels). **d** For recurrent gene pairs of *EPHA2-ATM* and *EP300-ATM*, dNdS ratios are shown in tumor types where the gene pairs were observed. Shaded and unshaded boxes represent the dNdS ratios with or without mutation contexts, respectively. **e** Differences in variant allele frequency (VAF) are illustrated for SYN and ANT gene pairs as measured in PanCancer datasets. **f** VAF differences of gene pairs as estimated in the corresponding tumor types are shown separately across the tumor types. *, **, *** represent *P* < 0.05, *P* < 0.01, and *P* < 0.001, respectively. **g** Differences in tumor mutation burden (TMB) are presented in a boxplot (log10 scale). **h** TMB differences are shown across tumor types examined. Asterisks indicates the level of statistical significance

The 312 genes examined using PanCancer profiles, were further evaluated across 20 tumor type-specific mutational profiles leading to the identification of 3870 gene pairs with tumor type-specific significant ED (see “Methods” for the selection criteria). The 3870 gene pairs with tumor type-specific ED, comprising 2523 missense, 587 truncating, and 760 indels, are presented for their dNdS ratios with or without mutation contexts (Fig. 3a) along with cdNS values (Fig. 3b). Detailed information of 3870 tumor type-specific gene pairs are available in Additional file 1: Table S3. The distribution of gene pairs across tumor types is illustrated (Fig. 3c). Tumor types with high tumor mutation burdens (TMB) including lung cancers and melanomas as well as those frequently showing microsatellite instability-high (MSI-H) such as colorectal, stomach, and endometrial cancers demonstrated a higher frequency of gene pairs with tumor type-specific ED. This suggests that the identification of gene pairs in tumor types with low mutation frequencies may not be exhaustive in current mutational profiles.

Among the gene pairs displaying the lowest cdNS values, indicating an ANT relationship, we noted that *RB1* and *RNF43* mutation contexts are antagonistic to *IDH1* missense and *APC* truncating mutations in gliomas and colorectal cancers, respectively (ranked 5th and 6th with cdNS scores of − 3.83 and − 3.76). The ANT relationship of *IDH1* and *RB1* mutations in brain tumors may have arisen due to the mutation preference of low- and high-grade gliomas that are respectively enriched with *IDH1* mutations and mutations in Rb pathways including *RB1* mutations [48]. In colorectal cancers, *RNF43* mutation contexts exhibit an ANT relationship with *APC* truncating mutations, particularly associated with MSI status, i.e., *RNF43* mutations are prevalent in MSI-H tumors [49] but *APC* mutations are relatively enriched in MSS cases [23]. Therefore, some of tumor type-specific ANT relationships may reflect mutations that are enriched in specific tumor subtypes, such as the grade of gliomas and the microsatellite instability (MSI) status of colorectal cancers. The highest cdNS value (cdNS = 9.72) was observed for kidney cancers for *TTN* missense mutations in the context of *TP53* mutations. Given that *TP53* mutations are associated with elevated genomic instability and a higher TMB and *TTN* is the largest gene with a higher propensity for mutations, this correlation may be indicative of elevated genomic instability. The second highest cdNS values (cdNS = 7.312) was noted for *NHLRC1* mutation contexts for *FAT1* truncating mutations in head and neck cancers. *NHLRC1* overexpression is known to stimulate cellular proliferation and invasion, possibly acting as AKT downstream effectors [50]. These phenotypic effects are similar to those associated with *FAT1* losses [51] indicative of their potential synergistic roles.

We also identified 92 gene pairs that occurred in more than one tumor type. The two most frequently occurring gene pairs were observed in three tumor types, *EPHA2-ATM* and *EP300-ATM* gene pairs. *EPHA2* and *EP300* mutations consistently show decreased dNdS ratios in the presence of *ATM* mutation contexts compared to those without *ATM* mutation contexts across three tumor types, respectively (Fig. 3d). While *ATM*-encoded peptides are known for their roles in activating the homologous recombination pathway for DNA repair [52, 53], recent findings suggest that *ATM* might also counteract the non-homologous end-joining (NHEJ) process by removing DNA-PKcs [54]. Furthermore, EphA2, encoded by *EPHA2* have a role in DNA repair through direct binding to DNA-PKcs, and thus, mutation of *EPHA2* can consequently affect the NHEJ pathway [55]. This functional connection with *ATM* losses could elucidate the observed ANT relationship *EPHA2* and *ATM* mutations across tumor types. Similarly, regarding *EP300* mutations, there is evidence suggesting a functional association where *ATM* losses lead to the failure of p300 protein phosphorylation [56], indicating functional redundancy between *ATM* losses and p300 deficiency.

We further investigated the association between ED of gene pairs and variant allele frequency (VAF), a metric indicating the clonality for mutations. For each gene pair, we calculated the median VAF of mutations for each gene. We then determined the difference between the median VAF values (ΔVAF), i.e., VAF of the partner gene minus VAF of the context gene, and compared them with respect to SYN and ANT relationship of gene pairs (Fig. 3e). Our analysis revealed a significant decrease and increase of ΔVAF values for SYN and ANT gene pairs (*P* = 1.5e − 27 and *P* = 3.9e − 23, *t*-test, respectively). A positive ΔVAF indicates that mutation contexts in ANT relationships are likely to be subclonal, while mutations of their partner gene are likely to be clonal. The difference in VAF between SYN and ANT gene pairs is consistently observed across tumor types (Fig. 3f). We also calculated the difference in TMB (ΔTMB) between genomes harboring both mutations and those with singleton mutations in paired genes. We observed that genomes harboring mutations in both genes of ANT gene pairs had a higher TMB as indicated by positive ΔTMB, while the opposite was observed for SYN gene pairs (*P* = 6.9e − 50 and *P* = 7.9e − 176, respectively) (Fig. 3g). This is also consistently observed across tumor types (Fig. 3h). Considering the specificity of tumor subtypes, we examined the relationship of VAF and TMB with SYN-ANT gene pairs in glioma subtypes and MSI status in colorectal cancers (Additional file 2: Supplementary Fig. 5), revealing a consistent association. These findings offer insights into the mutation acquisition in gene pairs with an ANT relationship, which are less likely to be fixed in cancer genomes due to negative selection. For example, the impact of subclonal, ANT mutation contexts on the acquisition of mutations in their partner genes may be relatively small compared to clonal SYN mutation contexts. The impact of negative selection is primarily attributed to clonal mutations rather than subclonal mutations [57]. Moreover, mutations in ANT relationship are tolerant in genomes with high TMB, which are relatively tolerant to genomic alterations.

### The association of genetic dependency and ED

We next investigated the relation between the impact of genetic perturbation of cancer cell lines with respect to their mutation configuration of genes in ED relationship. To explore the relationship between ED and genetic dependencies, we employed three databases of genetic screenings. One, AVANA [26] was based on CRISPR (clustered regularly interspaced short palindromic repeats). The other two, DEMETER2 [27] and DRIVE [28], were based on short hairpin or shRNA. Across each database, we collected data from cell lines harboring mutations targeted by gene silencing and further distinguished them into those with or without the mutation contexts. The effect size was determined by comparing the median of cell viability between these identified two groups of cell lines as previously described [22]. For example, the impact of *KRAS* silencing was assessed in cell lines with *KRAS* mutations and the effect size corresponding to *KRAS-NRAS* gene pairs was calculated by comparing the cell viability of those with and without *NRAS* mutations (≥ 2 cell lines for both groups were considered). We then determined the correlation between these effect sizes with cdNS scores of the corresponding gene pairs, as estimated in PanCancer mutational profiles, to evaluate the relationship between ED and genetic dependency.

It has been assumed that SYN and ANT mutation contexts will strengthen and lessen the impact of gene knockouts leading to decrease and increase of cell viability (here, compromising and rescuing effects on cell viability, respectively), since SYN gene pairs are implicated in oncogenic addiction and ANT gene pairs can rescue the gene silencing effects of partner mutations [22]. Thus, mutation contexts with high cdNS scores are expected to exhibit decreased effect sizes with knock-out mutations, while those with low cdNS scores are expected to exhibit elevated effect sizes. This expectation aligns with our observation that mutation contexts corresponding to SYN and ANT gene pairs showed substantial difference in effect size across databases for the selected gene pairs available for gene dependencies, i.e., AVANA (36 SYN–8 ANT pairs, *P* = 0.114; *t*-test), DEMETER2 (48 SYN–19 ANT pairs, *P* = 0.157; *t*-test), and DRIVE (35 SYN–14 ANT pairs, *P* = 0.403; *t*-test). The relationship was largely consistent for gene pairs examined in tumor type-specific datasets (Additional File 2: Supplementary Fig. 6).

**Fig. 4:**
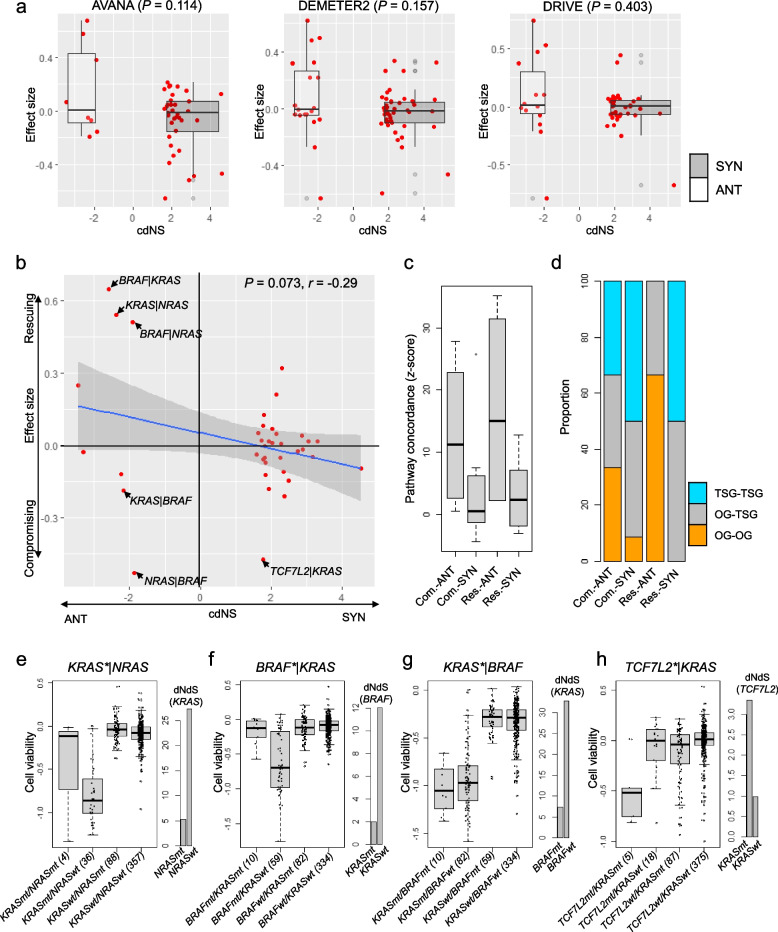
ED and genetic dependencies. **a** Effect sizes and dN/dS ratios for individual mutation pairs gene pairs are displayed across three databases of genetic dependencies (AVANA, DEMETER2, and DRIVE), with the *y*- and *x*-axis representing the mutation pairs and their respective dNdS ratios, respectively. The level of correlation and statistical significance by *t*-test is indicated. **b** Thirty eight mutation pairs gene pairs occurring more than once across databases are selected, and their effect sizes and dNdS ratios are presented. Four mutational categories are shown for rescuing and compromising effects (effect size > 0 and < 0, respectively) and synergistic and redundant/antagonistic effects (cdNS scores > 0 and < 0, respectively). **c** Boxplots depict pathway concordances for the four categories of mutation pairs. **d** The mutations in pairs are analyzed to determine whether they are oncogenes (OG) or tumor suppressor genes (TSG), and the abundance across the four categories of mutation pairs gene pairs is shown. **e–h** For selected examples of KRAS*-NRAS, BRAF*-KRAS, KRAS*-BRAF, and TCF7L2*-KRAS mutation pairs (where asterisk indicates the genes with knockouts), the cell viability of selected cancer cell lines with respect to their mutational states of gene members in the pairs are shown. Cell viability was used as available in AVANA database. Asterisks indicate knock-out genes with gene silencing. The dNdS ratios of mutations in genomes with or without the mutation contexts are also shown in barplots

We further compiled a list of 38 gene pairs that appeared more than once in three databases. These pairs showed a moderate yet substantial inverse relationship between ED and genetic dependencies, as indicated by cdNS scores and average effect size, respectively. (*r* =  − 0.29, *P* = 0.073; Fig. 4b, Additional file 1: Supplementary Table 4). These gene pairs were further categorized based on their impact on cell viability (either rescuing or compromising with effect sizes greater than 0 or less than 0, respectively) or ED (SYN and ANT with respect to cdNS). This classification resulted in four gene pair categories. We then estimated the pathway concordance of gene pairs (i.e., the number of molecular terms with gene members in the pair both included) and found that rescuing-antagonistic (Res.-ANT) gene pairs exhibited higher pathway concordance, followed by compromising-antagonistic (Com.-ANT) gene pairs (Fig. 4c). Therefore, the rescuing effects of gene pairs in ANT relationships can be largely ascribed to functional redundancy, as evidenced by a high level of pathway dependency, the degree of shared gene members within similar functional pathways. Further analysis based on whether the gene pairs represent the pairs of oncogenes or tumor suppressors showed that Res.-ANT pairs were more likely to be pairs of oncogenes (Fig. 4d). Thus, functionally redundant oncogene pairs appearing as ANT gene pairs can also provide rescuing effects in cell viability upon silencing of their paired mutations.

The cell viability is shown for selected gene pairs for one example of KRAS*-NRAS gene pairs (asterisk indicating silenced genes in cell lines) serving as an example of rescuing-antagonistic (Res.-ANT) gene pair (AVANA database, Fig. 4e). This specific case demonstrates that silencing mutant *KRAS* substantially impacts cell viability in *NRAS* wild type cells (*KRASmt/NRASwt*), while *NRAS* mutations counteract the effects of KRAS inhibition in double mutant cells (*NRASmt/KRASmt*; *P* = 0.296; *t*-test). This pattern was similarly observed when inhibiting *BRAF* mutants in both *KRAS*-mutant and -wildtype cells (*P* = 3.1e − 06; *t*-test, Fig. 4f). Interestingly, unlike the BRAF*-KRAS relationship, KRAS*-BRAF exhibits compromising effects, where *BRAF* mutations fail to mitigate the impact of *KRAS* knockout on *KRAS* mutant cells (Fig. 4g). It is presumed that the hierarchy of genes within the signaling cascade leads to varied effects on cell viability, i.e., in the Ras-Raf signaling pathway, mutations of *BRAF* that are downstream of Kras in the pathway, do not rescue *KRAS* mutant cells from *KRAS* knockouts. The cellular effects with respect to the mutation contexts are consistently observed across database (DEMETER2 and DRIVE, Additional File 2: Supplementary Fig S7).

### The association of the drug sensitivity and ED

**Fig. 5:**
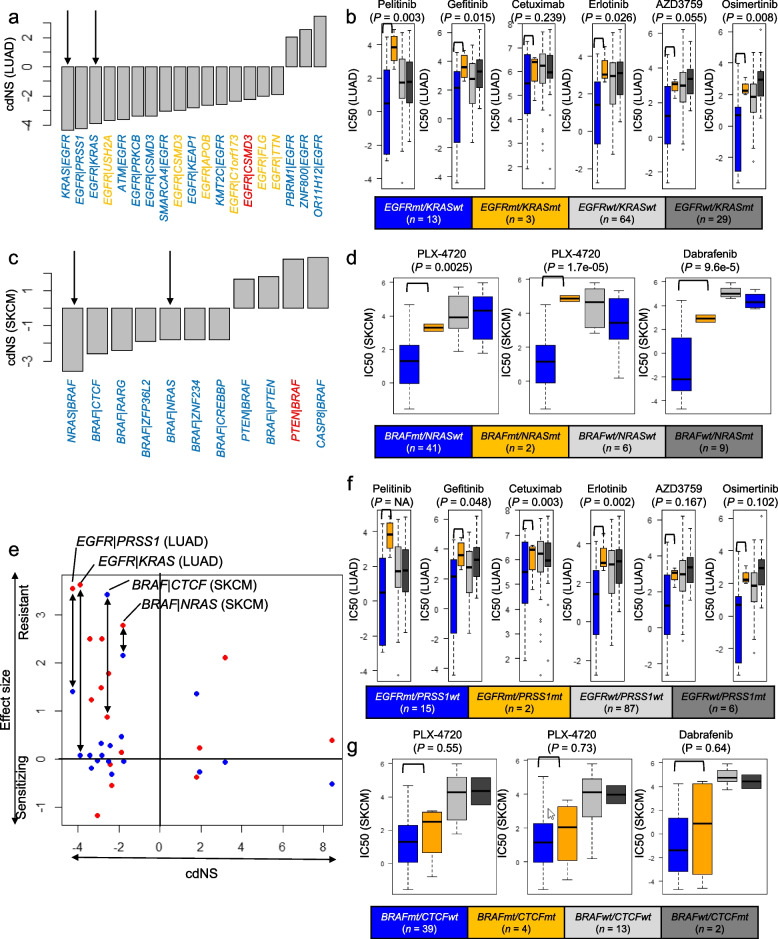
ED with pharmacological impact. **a** cdNS scores of gene pairs containing *EGFR* as gene members, as estimated in lung adenocarcinoma (LUAD) mutational profiles. Blue, orange, and red denote partner genes harboring significant ED for missense, truncating mutations, and indels, respectively, with corresponding mutation contexts. Two arrows indicate pairs of *EGFR* and *KRAS* mutations as significant ANT gene partners. **b** IC50 levels across six EGFR inhibitors are displayed for four types of LUAD cell lines based on the mutational states of *EGFR* and *KRAS* (*mt* and *wt* representing mutant and wild types, respectively). Drug names and significance levels estimated between *EGFRmt/KRASwt* (blue) and *EGFRmt/KRASmt* (orange) by *t*-test are illustrated. The number of cell lines is also indicated. IC50 values of celllines with *EGFRwt/KRASwt* (grey) and *EGFRwt/KRASmt* (black) are separately shown. **c** The cdNS scores are shown for *BRAF*-harboring gene pairs identified in melanoma (SKCM) mutational profiles. Arrows indicate ANT relationship between the *BRAF* mutations and *NRAS* mutations. **d** IC50 values with BRAF inhibitors are shown for SKCM cell lines with respect to the mutational status of *BRAF* and *NRAS* mutations. **e** The effect size (the differential of IC50 values of cell lines between those with or without the mutations paired with mutation contexts) is shown against cdNS scores of mutation pairs (*y*- and *x*-axis, respectively). Red and blue dots represent the effect size as estimated from GDSC and CCLE database. Pairs of two databases were linked by arrows for selected mutation pairs. **f,g** IC50 values are similarly shown for mutation pairs of *PRSS1-EGFR* and *CTCF-BRAF* for LUAD and SKCM celllines, respectively

We next examined whether the mutation contexts can influence the pharmacological effects of targeted agents according to their ED with the targeting genes. For the analysis, we focused on agents targeting *EGFR* and *BRAF* that are used in lung adenocarcinomas and melanomas (LUAD and SKCM), respectively. Among 279 gene pairs with tumor type-specific significant ED in LUAD, we selected 19 gene pairs including *EGFR* as either context genes or their partner genes. The cdNS values of 19 gene pairs are shown in Fig. 5a, highlighting ANT relationship of *EGFR* with *KRAS* mutations (i.e., cdNS scores of − 4.3 and − 3.9 for *KRAS* missense mutations with *EGFR* mutation context and for *EGFR* missense mutations with *KRAS* mutation contexts, respectively, arrows indicated in Fig. 5a). This ANT relationship suggests that *KRAS* mutations may compensate the *EGFR* mutations that are inhibited by EGFR inhibitors, thereby reducing the inhibitory effects. To demonstrate this, we examined IC50 (half maximal inhibitory concentration) levels of LUAD cell lines to EGFR inhibitors with respect to the mutational status of *EGFR* and *KRAS* as obtained in GDSC (Genomics of Drug Sensitivity in Cancer) [31] (Fig. 5b). Compared to *EGFR* wildtypes, *EGFR* mutant cell lines showed sensitivity to EGFR inhibitors only in the absence of *KRAS* mutations (*EGFRmt/KRASwt*) and the IC50 levels of cell lines harboring both *EGFR* and *KRAS* mutations (*EGFRmt/KRASmt*) were comparable to those of *EGFR* wild type cell lines across six EGFR inhibitors. A similar relationship was observed between drug sensitivity to BRAF inhibitors and the ED of *BRAF*-*NRAS* mutations in SKCM cell lines. For example, among 11 gene pairs including *BRAF* observed in SKCM, ANT relationship was noted with *NRAS* mutations (cdNS scores of − 3.6 and − 1.8, arrows in Fig. 5c). We also observed that *BRAFmt/NRASmt* SKCM cell lines showed comparable IC50 levels to those of *BRAF* wild type cell lines across three BRAF inhibitors (Fig. 5d). These findings suggest that for mutations of genes in ED can potentially alter drug sensitivity where the inhibitory impact of targeting agents is compromised by mutations that exhibit an ANT-relationship with targeted genes.

To demonstrate the relationship between ED and drug sensitivity, pharmacological effects were further evaluated across tumor type-specific gene pairs. For this analysis, effect sizes were estimated as the IC50 differential between tumor type-matched cell lines (i.e., those harboring mutations corresponding to the partner genes of mutation contexts) with or without mutations in genes of the mutation context. For example, IC50 differential between EGFR inhibitor-treated *EGFR*-mutant LUAD cell lines with or without *KRAS* mutation context (no less than two cell lines) was calculated as effect size across individual EGFR inhibitors. Then, they were compared with cdNS scores of *EGFR-KRAS* gene pair as evaluated in LUAD mutational profiles. We considered drugs whose targets correspond to the partner mutations, obtaining 18 gene pairs whose cdNS scores and effect sizes are available both in two database of GDSC and CCLE (Cancer Cell Line Encyclopedia) database [32]. The selected pairs are shown in scatter plots (Fig. 5e, those of GDSC and CCLE datasets shown in red and blue, respectively). Substantial level of correlation was observed although not in significant levels (*r* =  − 0.25, *P* = 0.146). Among the gene pairs, we observed other candidates of rescuing pairs of *PRSS1* and *CTCF* mutations occurring with *EGFR* and *BRAF* mutation contexts in LUAD and SKCM, respectively. When *PRSS1* and *CTCF* mutations were examined for their impact on IC50 of cell lines treated with EGFR and BRAF inhibitors, we observed similar compromising effects, e.g., their co-mutations with targeted mutations showed an elevation in the IC50 of the cell lines (Fig. 5f, g). This suggests that *PRSS1* and *CTCF* mutations exhibit compensatory effects comparable to *EGFR* and *BRAF* mutations in the therapeutic response to EGFR and BRAF inhibitors in LUAD and SKCM cell lines.

## Discussion

In this study, we adopted evolutionary measures based on dNdS (nonsynonymous-to-synonymous) ratios and their derived cdNS (context-dependent dNdS ratios) scores to assess the ED (evolutionary dependency) of gene mutation pairs in cancer genomes. Although positive and negative selective pressures have influenced the development of acquired mutations, ultimately shaping the mutation landscape of cancer genomes [21, 58, 59], the relationship between these mutations remains largely unknown. One assumption underlying the study is that the selection of mutations in cancer genomes is an evolutionarily constrained and context-dependent process leaving genomic footprints in snapshots of cancer genomes. Thus, we suggest that the comparison of dNdS ratios of mutations in relation to the mutation context, can serve as a measure of ED. Previous studies have proposed similar methodologies, e.g., the comparison of two dNdS ratios between clonal and subclonal mutations in lung cancers holds potential clinical significance [60]. However, our extensive analyses on a broader set of genes and tumor types uncovered a range of functional and cellular aspects of gene pairs influenced by ED across various tumor types. Our newly introduced ED measures referred to as cdNS scores, encapsulate the essence of dNdS ratios, i.e., they not only categorize the types of gene pairs (synergistic and antagonistic pairs, SYN and ANT) but also offer quantitative assessments of ED between two genes. Upon applying ED to known gene pairs identified by genomic distribution (517 CO-ME gene pairs as co-occurring and mutually exclusive ones) [25], we observed a strong correlation between the CO and ME categories of CO-ME gene pairs and cdNS-based SYN and ANT pairs. Furthermore, the variability of ED for the observed gene pairs also underscores the necessity for quantitative metrics like cdNS scores. It should also be noted that the ANT gene pairs in PanCancer analyses might appear between two tumor type-specific genes simply due to their differing tumor type specificities. Although we have used weighting factors to mitigate this impact [25], future research should focus on further minimizing the influence of tumor type-specific effects when identifying PanCancer-ANT gene pairs.

Utilizing cdNS scores as novel ED measures, we evaluated gene pairs in cancer genomes with respect to other molecular and genomic features including VAF (variant allele frequencies) and TMB (tumor mutation burdens) as well as genetic dependency and pharmacological perturbations. The quantitative nature of cdNS scores reveals significant variation in the degree of ED, often exceeding tenfold positive or negative selective pressure for mutational acquisition according to the mutation contexts. This relationship often leads to the changes in the cell survival of cell lines with knock-outs, which are consistent with the types of ED where ANT and SYN gene pairs tend to show rescuing or compromising effect on the survival of cells. For example, a *KRAS*-*NRAS* ANT gene pair with low cdNS score rescues cell survival, indicating that *NRAS* mutation contexts rescue the impact of *KRAS* knockouts on *KRAS*-mutant cell lines. In contrast, *KRAS* mutation contexts decreased the survival of *TCF7L2*-mutant cells with TCF7L2 knockout, suggestive of potential synthetic lethality [61]. Of note, the relationship of ED and genetic dependency depends on the hierarchy of gene members in the signaling cascade [62] as shown with the relationship of *BRAF* and *KRAS*. For example, the *KRAS* mutation contexts showed rescuing effects on survival of *BRAF*-mutant cells with *BRAF* knock-outs, whereas *BRAF* mutation contexts fail to rescue the effect of *KRAS* knock-outs on *KRAS*-mutant cells. Given the hierarchy within the receptor tyrosine kinase signaling pathway including Ras-Raf, mutation contexts of Raf exert relatively minor effects on the mutations and knock-outs of its upstream Ras, while Ras mutations exhibit greater influence as upstream mutation contexts [63].

The dNdS ratio-based ED can also highlight resistance-conferring mutations to targeted agents such as *KRAS* and *NRAS* mutations for EGFR and BRAF inhibitors, respectively [64, 65]. This suggests that ED may assist in selecting patients who might benefit from targeted agents or predicting disease recurrence [15]. For example, while the impact of *KRAS* and *NRAS* mutations on the EGFR and BRAF inhibitors have been well-recognized, the ED-based analyses further demonstrated genes with similar ED and pharmacological effects with *KRAS* and *NRAS* mutations. We observed that *PRSS1* and *CTCF* mutations in lung adenocarcinomas and skin melanomas show similar resistance-conferring effects to *KRAS* and *NRAS* mutations when treated with EGFR and BRAF inhibitors. *PRSS1* encodes pancreatic serine proteinase, trypsin-1 whose expression has been associated with the sensitivity to EGFR inhibitors of cetuximab in colorectal cancers, and its inhibition reduces the tumor growth [66]. *CTCF* encoding CCCTC-binding factor mediates transcriptional regulation of *DUSP6* and when inactivated, can activate MAPK signaling [67], which explains the resistance mechanisms to BRAF inhibitors with *CTCF* losses.

One limitation of our study is that ED analyses are limited to somatic mutations, whereas cancer genomes include other types of genomic alterations, such as DNA copy number alterations and chromosomal rearrangements [68]. One important assumption of dNdS ratios is that alterations that can be distinguished into non-neutral *vs.* neutral changes. Compared to nonsynonymous-*vs.*-synonymous mutation calls, other types of genomic alterations cannot be readily distinguished as non-neutral or neutral because large-scale chromosomal changes often involve many genes and have non-binary nature (e.g., copy number states). The heterogeneity of cancer genomes sharing multiple types of alterations in single locus also complicates the determination of functionality for large-scale genomic alterations [69]. Nevertheless, the consideration of genomic alterations in addition to somatic mutations will be essential for gaining a comprehensive understanding of the mutational landscape and identification of potential interactions between various types of genomic alterations. Another assumption underlying our study is that mutations in cancer genomes do not simultaneously occur, so that the first mutational hit in the mutation pair provides the ED for the second hit. However, kataegis is an example that violates this assumption, because a number of mutations occur simultaneously in localized genomic regions [70]. Since this genomic event has unique properties such as local adjacency and common mutation signatures, it may be feasible that somatic mutations can be categorized further [71] and subject to cdNS-based ED analyses. In addition, it should be considered that our cell line-based results might reflect different ED compared to those observed in primary tumors, as in vitro system lacks immune cells and has abundant cultural resources.

In our study, mutation contexts were established using genomes containing non-silent mutations of genes, without discrimination based on types of mutations. Thus, there is still a potential for considering distinct mutation types within mutation contexts and incorporating functionality measures to refine the ED of mutation pairs. For example, hotspot missense mutations [72] and those categorized based on functional measures [3] can be further considered in evaluating dNdS ratios and cdNS scores. Our analysis of three cell lines harboring mutations on both *KRAS* and *EGFR* (Fig. 5b), all *KRAS* mutations are located on known hotspots (12th and 13th amino acid residues), while *EGFR* mutations were near or outside the known hotspots mutations [72] raising a concern related with the functionality of observed gene pairs. Although our current methods do not integrate site-level information, it prompts further investigation into whether integrating functionally filtered mutational profiles could refine ED relationship. However, it is important to note the limited availability of genomes with mutation contexts since the mutations occur in a small fraction of cancer genomes. The limited number of available datasets may introduce potential biases in this type of analysis particularly when focusing on the pairs of genomic events (e.g., double mutants) that are often extremely scarce in public resources such as the CCLE database. This scarcity inadvertently highlights molecular pathways whose genes frequently harbor somatic mutations, such as the Ras pathway, underscoring the need for additional resources to draw robust conclusions. In addition, one observation of our study is the prevalence of tumor type-specific gene pairs among those with high tumor mutational burden (TMB). While it is possible that the hypermutated genomes may harbor an increased number of functional gene pairs, it is still conceivable that the current list of gene pairs may not be fully comprehensive and could benefit from larger mutation databases in the future [3, 73].

## Conclusions

In summary, our study provides evolutionary perspectives on functional gene pairs in cancer genomes by employing dNdS ratio-based ED analyses. The dNdS ratio as well as novel ED measure of cdNS score was able to distinguish SYN and ANT gene pairs along with ED reflecting their functionality. The ED of gene pairs were examined for associations with other evolutionary and phenotypic features, revealing that ED of gene pairs represents unique functional signatures of mutations in cancer genomes. By employing cell line-based studies of genetic dependencies and pharmacological perturbation, ED guided the selection of candidates underlying synthetic lethality and drug-sensitizing or resistant mutations.

### Supplementary Information

Additional file 1: Table S1. Gene used for ED analyses. Table S2. PanCancer gene pairs with ED. Table S3. Tumor type-specific gene pairs with ED. Table S4. ED and genetic dependencies. Table S5. Effect size of targeted agents with ED of gene pairs

Additional file 2: Supplementary Fig. S1. The subsampling analysis of mutation contexts. Supplementary Fig. S2. ED of known gene pairs. Supplementary Fig. S3. ED in panel sequencing data. Supplementary Fig. S4. ED of mutations conserved across Ras mutation contexts. Supplementary Fig. S5. VAF and TMB of SYN and ANT gene pairs in tumor subtypes. Supplementary Fig. S6. Genetic dependency and tumor type-specific gene pairs. Supplementary Fig. S7. Cell viability of synergistic and antagonistic mutation pairs

## Data Availability

Somatic mutation calls of TCGA consortium and related information were downloaded from TCGA data portal [74]. Panel-based mutational profiles were downloaded from cBioPortal link [75]. Cancer-related genes were used as Cancer Census genes [76]. dvdscv R packages were downloaded from Github database [77]. Gene dependency scores were obtained from Dependency Map (DepMap) gene essentiality screenings [78]. Specifically, AVANA scores (18q3_v3, 17 k genes across 485 cell lines) and DEMETER2 scores (v2, 17 k genes across 712 cell lines) were obtained along with DRIVE database (7.8 k genes across 398 cell lines, version5, May 2019). Drug response data of cancer cell lines were obtained from GDSC (Genomics of Drug Sensitivity in Cancer) database as a combined set of two dataset versions (GDSC1 and GDSC2) [79]. Other pharmacological cancer cell line data were obtained from CCLE (Cancer Cell Line Encyclopedia) databases [80]. The R code used to derive the cdNS scores are available (https://github.com/TkimLab/cdNS) [81].
