# Supplementary material for: Evolutionary dependency of cancer mutations in gene pairs inferred by nonsynonymous-synonymous mutation ratios
Source: Genome Med. 2024 Aug 19;16:103. doi: 10.1186/s13073-024-01376-7 (PMC11331682; doi:10.1186/s13073-024-01376-7)
Supplement: Supplementary file 2 — Additional file 2: Supplementary Fig. S1. The subsampling analysis of mutation contexts. Supplementary Fig. S2. ED of known gene pairs. Supplementary Fig. S3. ED in panel sequencing data. Supplementary Fig. S4. ED of mutations conserved across Ras mutation contexts. Supplementary Fig. S5. VAF and TMB of SYN and ANT gene pairs in tumor subtypes. Supplementary Fig. S6. Genetic dependency and tumor type-specific gene pairs. Supplementary Fig. S7. Cell viability of synergistic and antagonistic mutation pairs [file 13073_2024_1376_MOESM2_ESM.pdf]

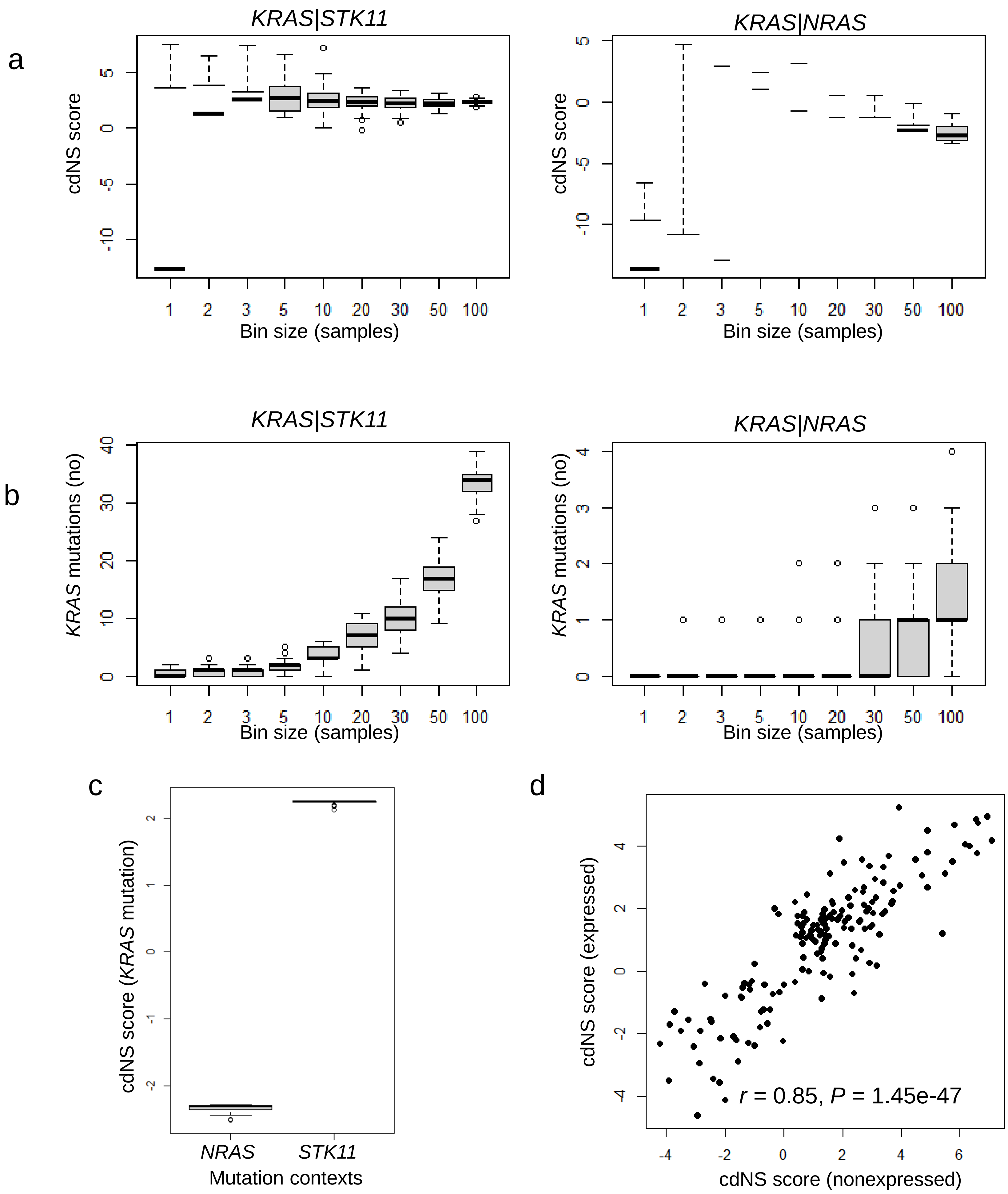

Supplementary Fig. S1. The subsampling analysis of mutation contexts. a For two gene pairs (*KRAS-STK11*/SYN and *KRAS-NRAS*/ANT), cdNS scores (*y*-axis) were estimated in 100 subsampling tests datasets in a range of 1 to 100 genomes with mutation contexts (mutation context+) (*x*-axis). **b** Relatively robust cdNS scores were observed up to 10 and 50 mutation context+ genomes where 3 and 1 *KRAS* mutations (median) were observed for *KRAS-STK11* and *KRAS-NRAS* pairs, respectively. **c** cdNS scores of KRAS mutations were estimated in 100 subsampled datasets with an equal distribution of mutation context+ and context- genomes set at 100 each. **d** cdNS scores of 82 PanCancer mutation pairs were assessed across genomes with mutation context+, divided equally between those expressed for genes corresponding to mutation context (expressed) and those not (nonexpressed).

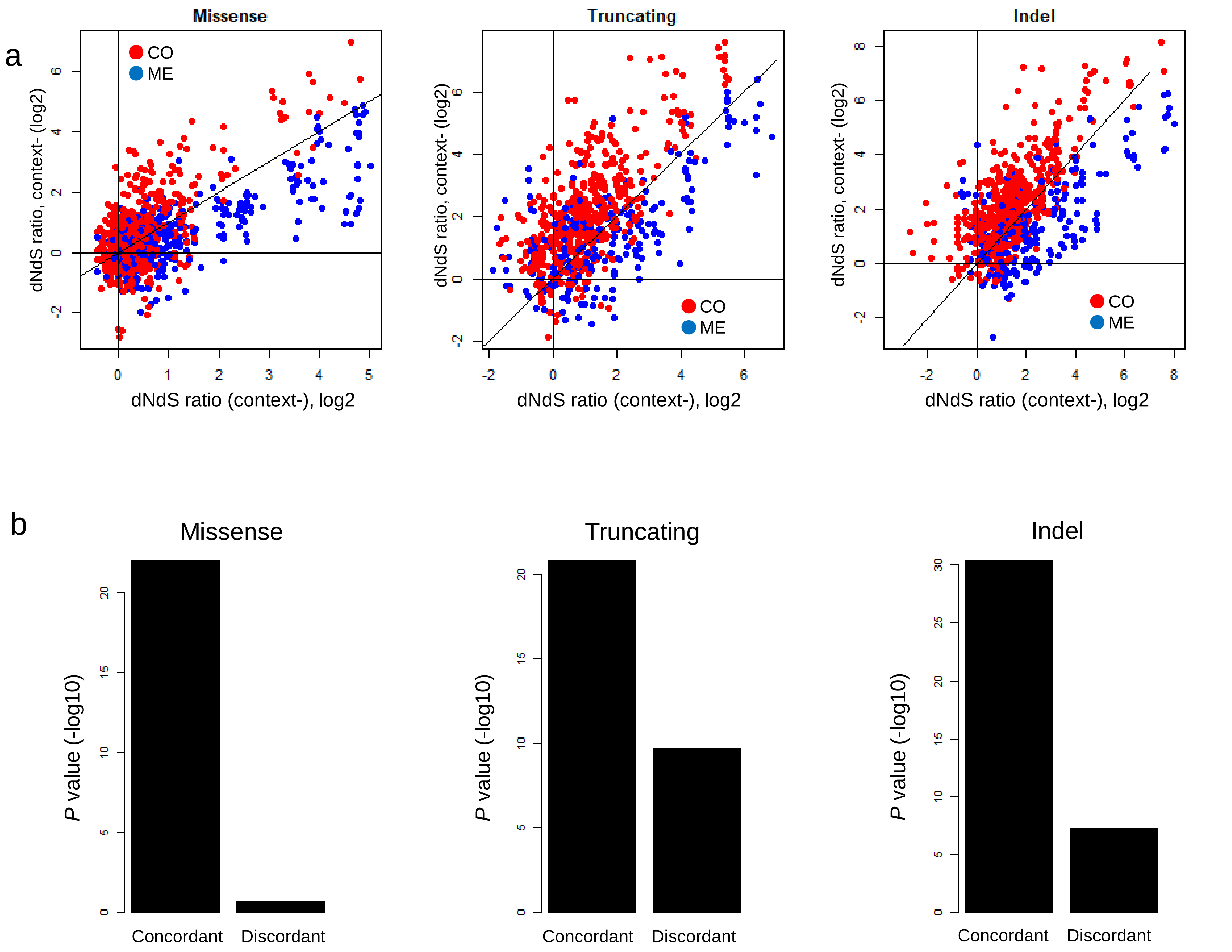

Supplementary Fig. S2. ED of known gene pairs. a Scatter plots show two dNdS ratios for 517 known mutation pairs (dNdS context+ and context- on the *y*- and *x*-axis, respectively) with mutation pairs categorized based on functional relationships (red and blue for co-occurrences (CO) and mutual exclusivity (ME) representation, respectively). Scatter plots are separately shown for missense, truncating, and indel mutations. **b** Concordant and discordant pairs are analyzed for their enrichment in cancer-related genes (Cancer Census genes), with the significance of enrichment evaluated using Fisher's exact test

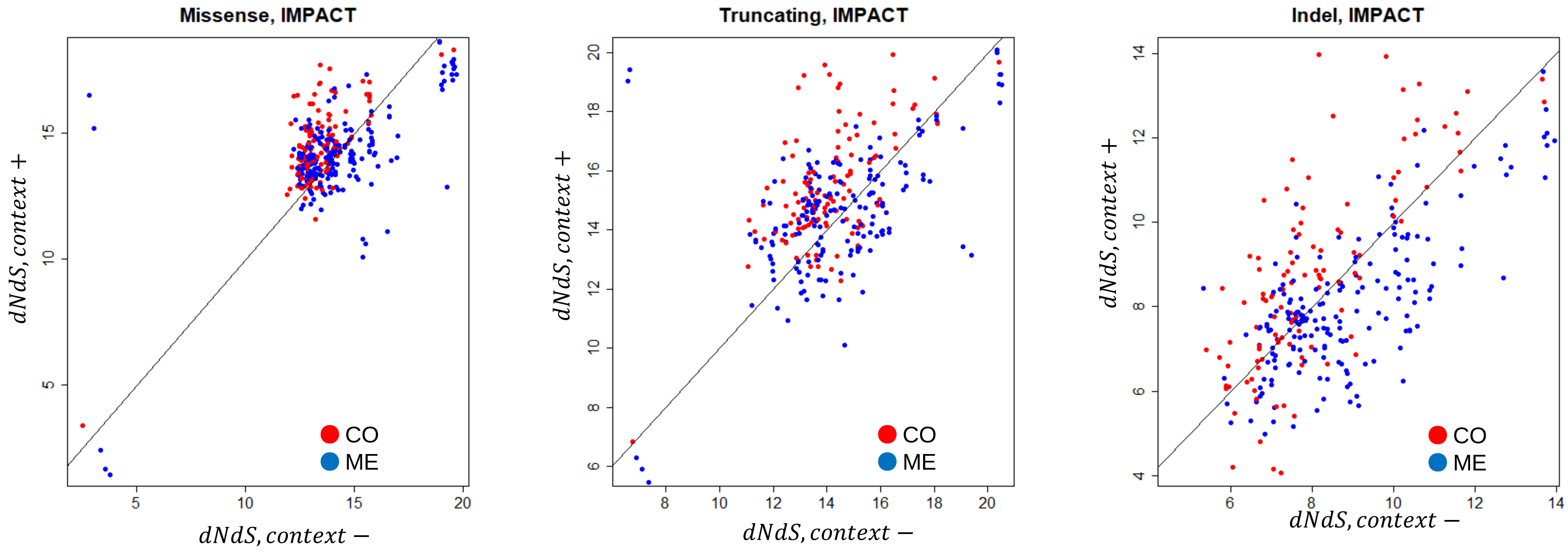

Supplementary Fig. S3. ED in panel sequencing data. Context-dependent dNdS ratios are shown as ED for three types of mutations (missense, truncating and indels) for MSK-IMPACT datasets. dNdS ratios of genes without and with the mutational contexts (dNdS, context- and context+) are shown in x- and y-axis, respectively.

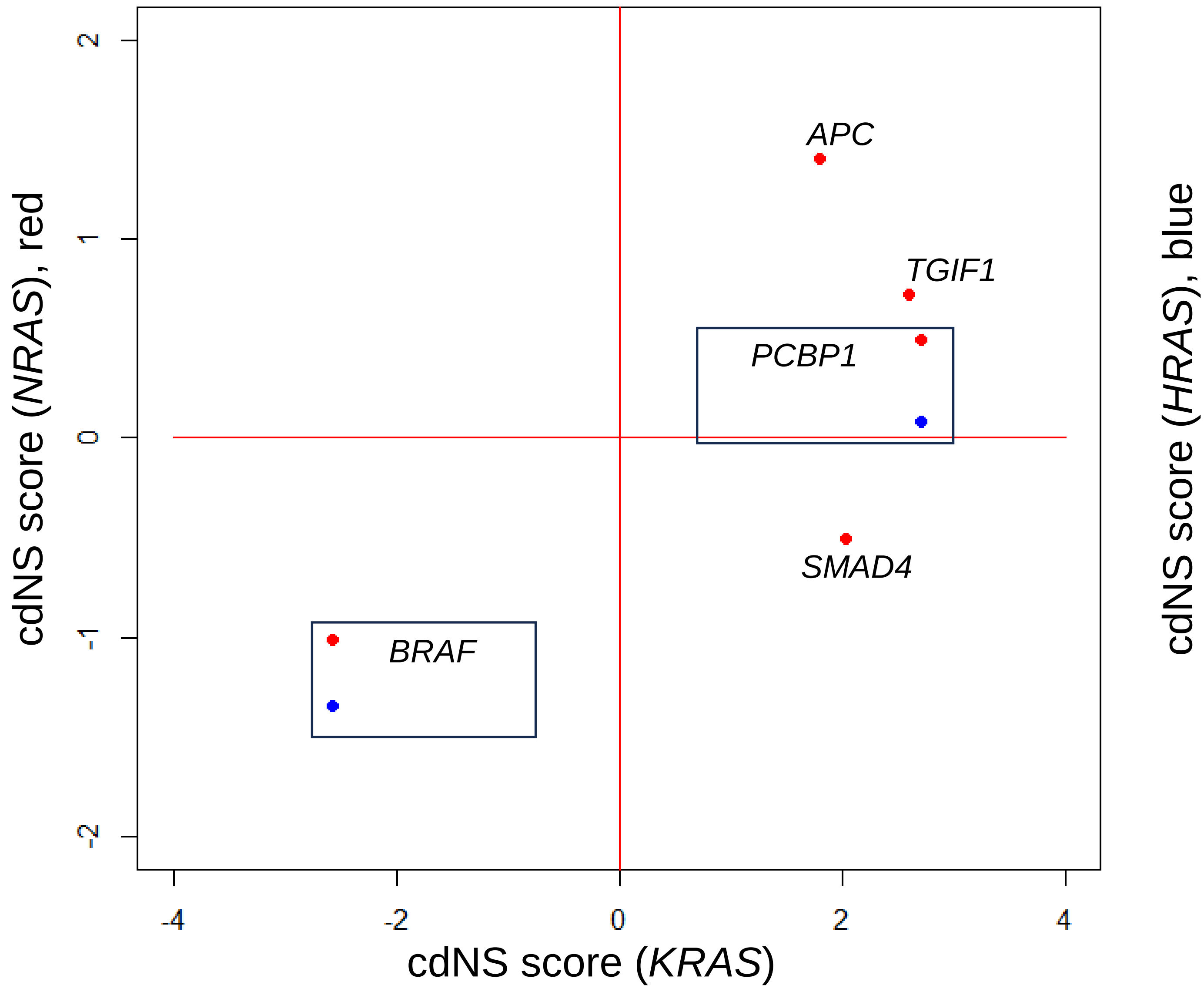

Supplementary Fig. S4. ED of mutations conserved across Ras mutation contexts. The ED, depicted as cdNS scores, is displayed for genes exhibiting ED with *KRAS* mutations (as depicted in the main Figure 2), across three Ras genes: *KRAS, NRAS*, and *HRAS*. The cdNS scores under *KRAS* mutation contexts are represented on the *x*-axis, while those under *NRAS* (in red) and *HRAS* mutation contexts (in blue) are represented on the *y*-axis. Our observation reveals a consistent pattern of cdNS scores across available genes, emphasizing the association of ANT relationship with negative cdNS scores, particularly noted with *BRAF* mutations within *KRAS* mutation contexts, which are commonly observed within *NRAS* and *HRAS* mutation contexts.

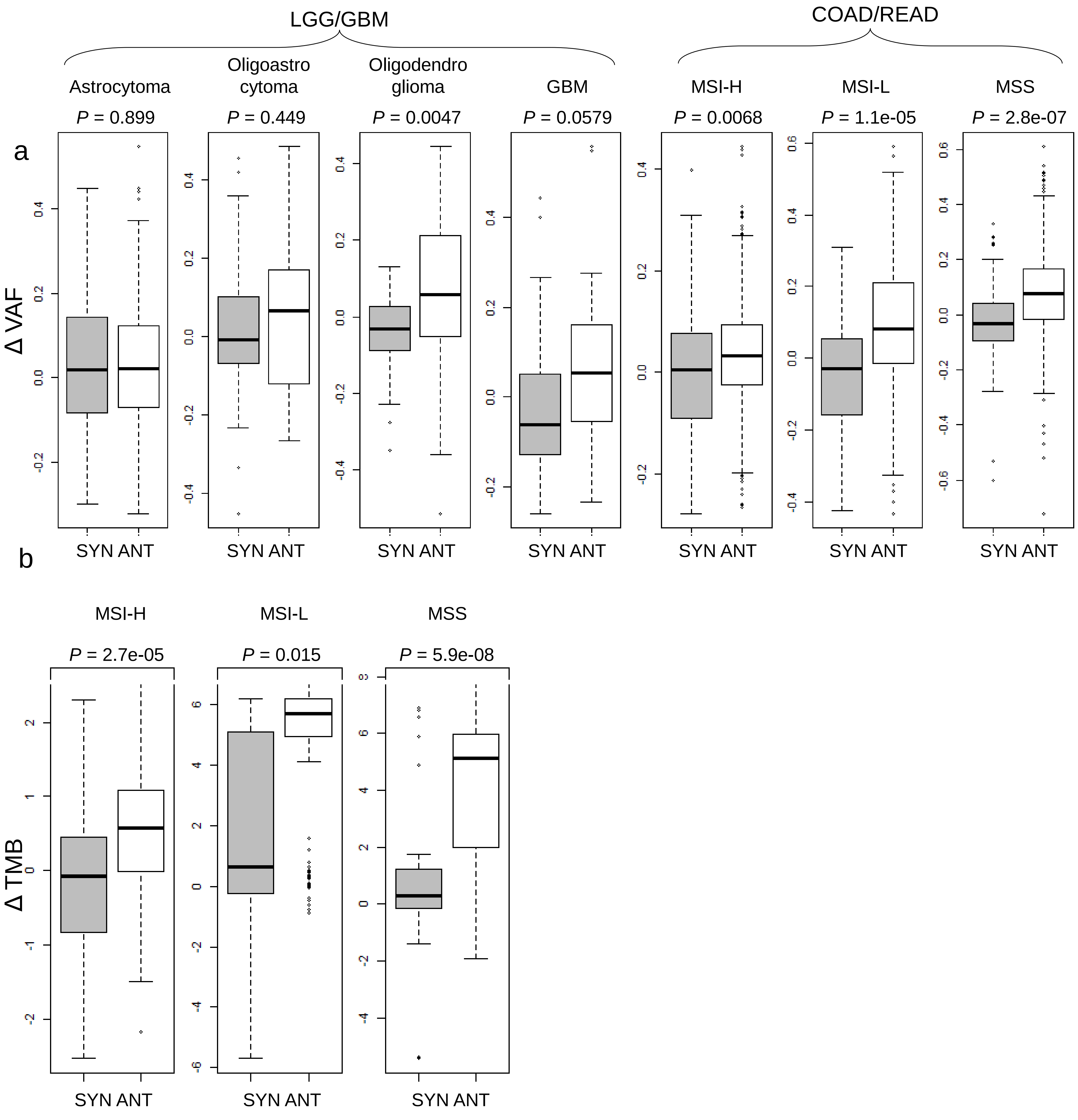

Supplementary Fig. S5. VAF and TMB of SYN and ANT gene pairs in tumor subtypes. a The figure presents the disparities in VAF (Δ VAF) between SYN and ANT gene pairs across four glioma subtypes, encompassing low-grade gliomas (LGG; including astrocytoma, oligoastrocytoma, and oligodendroglioma) along with glioblastoma multiforme (GBM). Similarly, it demonstrates the differences in VAF (Δ VAF) based on MSI (Microsatellite Instability) status in colorectal cancers (COAD/READ). **b** For TMB, the figure depicts the discrepancies in TMB (Δ TMB) for SYN and ANT gene pairs across three MSI-based COAD/READ genomes. TMB analyses omit LGG/GBM tumors due to the unavailability of gene pairs for TMB assessment in LGG cases.

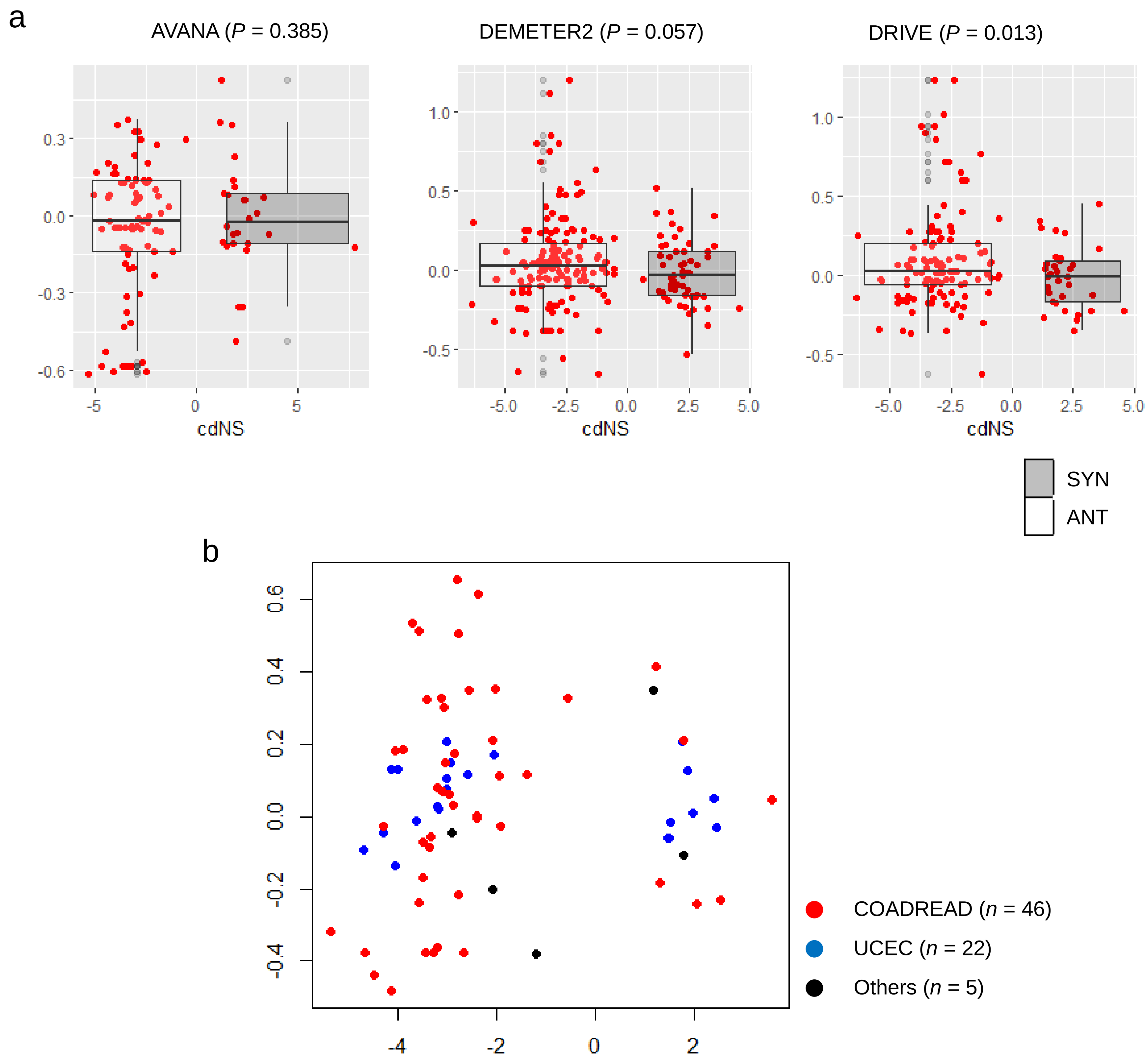

Supplementary Fig. S6. Genetic dependency and tumor type-specific gene pairs. a The effect size (*y*-axis) are shown with respect to ED types of SYN and ANT gene pairs across three database (AVANA, DEMETER2 and DRIVE). **b** For tumor type-specific genes pairs observed more than one of three databases are shown for their averaged effect size (y-axis) and cdNS scores (x-axis). We note that more than 93.1% of filtered gene pairs are derived of two tumor types of COAD/READ and UCEC.

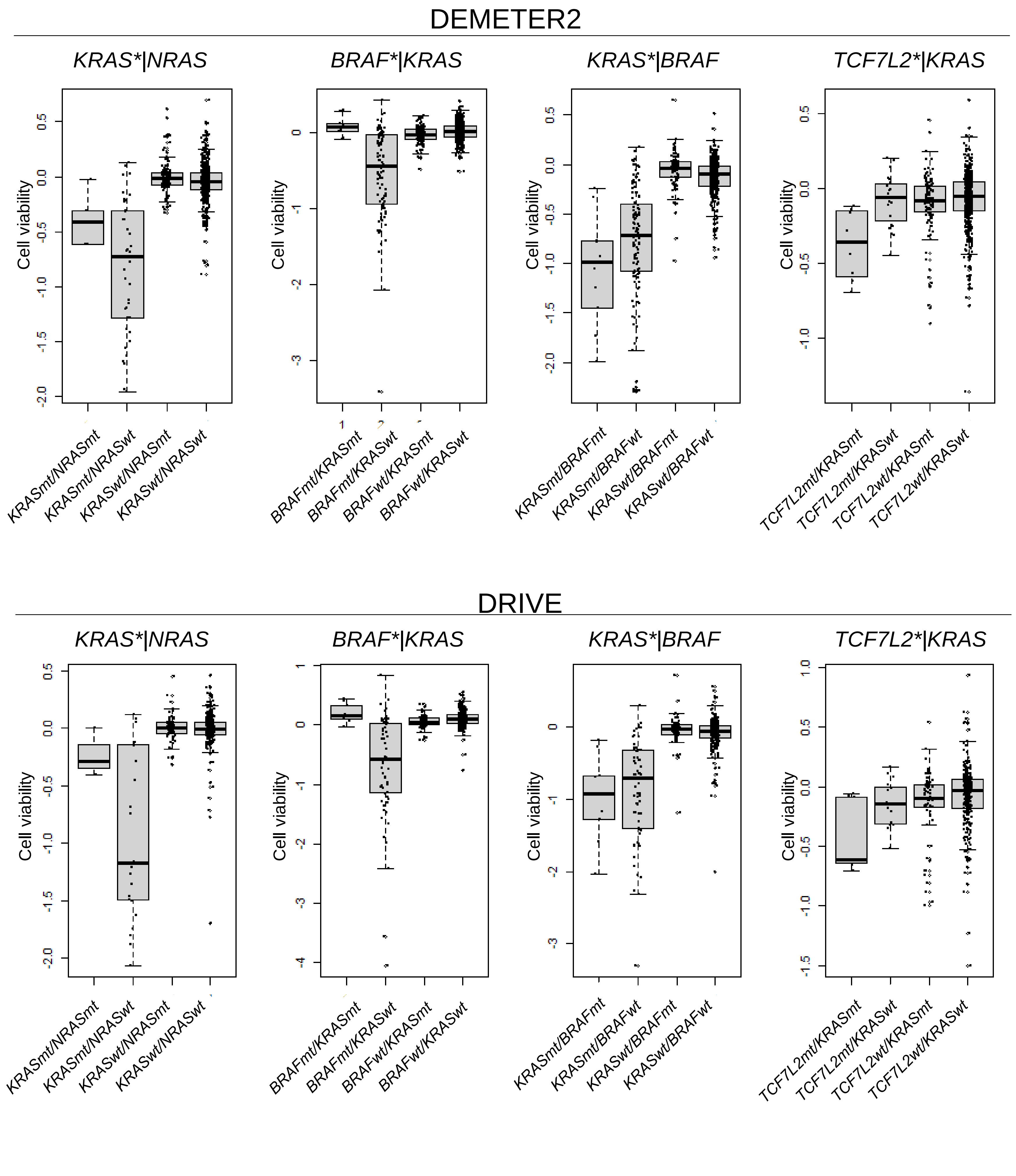

Supplementary Fig. S7. Cell viability of synergistic and antagonistic mutation pairs. For four mutation pairs of *KRAS*-NRAS, BRAF*-KRAS, KRAS*-BRAF* and *TCF7L2*-KRAS*, the cell viability of cells harboring different gene mutations are for DEMETER2 and DRIVE database. Asterisk indicates genes silenced .
